# Establishment and Characterization of an Embryonic Cell Line from S*arconesiopsis magellanica*

**DOI:** 10.1673/031.013.13001

**Published:** 2013-11-21

**Authors:** Mónica Cruz, Felio J. Bello

**Affiliations:** Laboratorio de Entomología Médica y Forense, Escuela de Medicina y Ciencias de La Salud, Universidad Del Rosario, Calle 63D 24-31, Bogotá, Colombia

**Keywords:** cell cultures, cell morphology, cytogenetic, isozyme patterns, RAPD-PCR

## Abstract

*Sarconesiopsis magellanica* (Le Guillou) (Diptera: Calliphoridae) is a necrophagous fly that is important in both human and veterinary medicines. This insect has been registered in Colombia as a biological indicator in estimating post-mortem interval. Insect cell cultures are an important biotechnological tool for basic and applied studies, and cell cultures derived from *S. magellanica* embryonic tissues are described in this study. *S. magellanica* embryonated eggs were taken for tissue explants. These were seeded in L-15, Grace/L-15, Eagle MEM, MM, VP12, MM/VP12, and Schneider culture media. The morphological, cytogenetic, biochemical, and molecular characteristics of the cell cultures were examined. Cell growth was achieved in the L15, Grace/L15, and Schneider culture media, and the confluent monolayers were obtained 8, 10, and 19 days after the embryonated eggs were explanted. However, the Schneider medium was the most efficient to develop the subcultures, and 21 passages have been maintained. The cell morphology of the primary cell cultures was initially heterogeneous, but in the confluent monolayer and in the subcultures there was greater cell morphology uniformity, fibroblastoid types being predominant. Cultured cells had a chromosomal number of 12, and the karyotypic complement consisted of five pairs of somatic chromosomes and one sexual pair. The cell culture isozyme patterns of *S. magellanica* coincided with adult samples from the same species. The molecular analysis, using RAPD-PCR, demonstrated the authentication of the cell cultures of this fly and their differentiation from other cultures derived from two sand flies species. This cell line is a new *in vitro* model that will be used in biomedical and biotechnological studies.

## Resumen

*Sarconesiopsis magellanica* (Le Guillou) (Diptera: Calliphoridae) es una mosca necrófaga de importancia en medicina humana y veterinaria. Este insecto se ha registrado en Colombia como indicador biológico ya que puede ser utilizado en la determinación del intervalo post-mortem. Por otra parte, los cultivos de células de insectos son una herramienta biotecnológica importante para estudios básicos y aplicados. Aquí se describen, por vez primera, cultivos celulares derivados de tejidos embrionarios de *S. magellanica*. Huevos de *S. magellanica* fueron tomados para los explantes. Estos fueron sembrados en los medios de cultivo: L-15, Grace/L-15, Eagle MEM, MM, VP12, MM/VP12 y Schneider. Las características morfológicas, citogenéticas, bioquímicas y moleculares de los cultivos celulares fueron examinados. El crecimiento celular se logró en los medios de cultivo L15, Grace/L15 y Schneider, obteniendo monocapas confluentes a los 8, 10 y 19 días después de realizado el explante. Sin embargo, el medio Schneider fue el más eficiente para desarrollar los subcultivos y 20 pases se han realizado hasta la fecha. La morfología de las células de los cultivos celulares primarios fue inicialmente heterogéneo, pero en la monocapa confluente y en los subcultivos, hubo una mayor uniformidad de la morfología celular, siendo los tipos fibroblastoides los más predominantes. Las células cultivadas exhibieron un número cromosómico de 12 y el complemento cariotípico consistió de 5 pares de cromosomas somáticos y 1 par sexual. Los patrones de cultivo celulares de isoenzimas de *S. magellanica* coincidieron con las muestras de adultos de la misma especie. El análisis molecular, utilizando RAPD-PCR, demostraron la autenticación de los cultivos de células de esta mosca y su diferenciación de otros cultivos derivadas de dos especies de mosquito. Esta línea celular es un nuevo modelo *in vitro* que podrá ser utilizada en estudios biomédicos y biotecnológicos.

## Introduction

*Sarconesiopsis magellanica* (Le Guillou) (Diptera: Calliphoridae) is a necrophagous and hemisynanthropic fly (Pinilla et al. 2012). This species is important in human and veterinary medicine because its larvae cause myiasis in some vertebrates, including humans (Stevens 2003; Visciarelli et al. 2003), and it is a potential mechanical vector for pathogens such as viruses, bacteria, fungi, protozoa, and helminths (Förster et al. 2003; Mendonça et al. 2012). Furthermore, this fly is important in medical-legal investigations because it can be used in the estimation of the post-mortem interval by participating as the first colonizer of decomposing corpses (Segura et al. 2009).

*S. magellanica* has been reported in South America, specifically in Colombia, Ecuador, Perú, Bolivia, Chile, and Argentina (Pape et al. 2004). Mariluis and Mulieri (2003) described this insect as being specially adapted to the heights, due to its location in areas over 900 m a.s.l. In Colombia*, S. magellanica* was recorded in the departments of Antioquia, Boyacá, Cundinamarca, and Norte de Santander (Pape et al. 2004), particularly in places located from 1,200 to 3,100 m a.s.l.

Since the early twentieth century, some researchers tried to obtain *in vitro* insect cells, but it was not possible until Grace (1962) established the first cell line from ovaries of a lepidopteran, the Australian emperor moth, *Antheraea eucalypti* (Grace 1962). Since then, more than 500 insect cell lines have been developed from 120 different insect species, but most of them are from Lepidoptera and Diptera (Lynn 2007). Insect cell lines have contributed to progress in physiological studies on the species from which they have been derived. These cell cultures have also been used in several fields of study, such as virology, immunology, developmental biology, and research on biopesticides (Zhang et al. 2011). Insect cell lines are considered as a potential resource in molecular biology because of its use in a wide range of applied research (Iwanaga et al. 2009). Likewise, these cultures have been very useful in the study of host-parasite interactions (Côrtes et al. 2011), in the propagation of specific pathogens (Takahashi et al. 1980), and in the baculovirus- insect cell expression system (Pan et al. 2010). This system has application for basic scientific research activities, diagnostic activities, and development and production of vaccines (Senger et al. 2009). Therefore, the usefulness of insect cell lines for protein production has grown from laboratory-scale experimental work to industrial applications (Elias et al. 2007).

Given the wide applicability of the cell cultures originating from different insect species, and taking into account that they are not useful as substrates for the same purposes in the several fields of research (Lynn 2007), because they tend to differ in their capacities to replicate viruses and express recombinant proteins (Elias et al. 2007), it is necessary to establish new insect cell lines (Zhang et al. 2011).

The present study describes the morphological, karyological, isozymatic, and molecular characteristics of a new cell line derived from *S. magellanica* embryonic tissues.

## Materials and Methods

### Obtaining biological material

Embryonated eggs from *S. magellanica* were taken from a colony previously established in the laboratory of the Medical and Forensic Entomology department of Rosario University, Bogotá, Colombia (4° 36′ 43″ N, 74° 04′ 07″ W, 2,600 m a.s.l.), which is the geographical center of Colombia. The study was carried out from March 2011 to June 2012. These flies were maintained in the insectary at 24 ± 1° C average temperature, 60 ± 5% RH, and a 12:12 L:D photoperiod. A flask with a cotton pad soaked in a sugar solution (15% sucrose) was used in each cage as a carbohydrate source. Pig liver was used as a substrate and a protein source to induce the oviposition of the adult females.

### Primary culture initiation

For each explant of embryonated tissues, approximately 200 eggs were placed in a Petri dish. After an incubation period of 20 to 22 hr, the eggs were refrigerated for 3 hr and then incubated at 37° C for 2 hr. The eggs were then placed into a 50 mL centrifuge tube where their surfaces were sterilized according to the procedure described by Figueroa et al. (2007) with some modifications. Initially, the eggs were rinsed with a 0.5% solution of sodium hypochlorite, and the tube was stirred repeatedly for 5 min. The solution was then removed, 5% formaldehyde was added, and the tube was continuously stirred for 5 min. Finally, the eggs were washed three times with sterile distilled water containing 1% antibiotics (penicillin /streptomycin). The eggs were placed into a Ten Broeck homogenizer (Pyrex, Corning, www.corning.com) where they were mechanically disrupted and dissociated with 3 mL trypsin for 4 min at 37° C. Dissociation was stopped by adding 5 mL of selected growth medium. The embryonic tissues were transferred to a centrifuge tube and centrifuged at 400 × g for 7 min. The pellet was re-suspended in 5 mL of the growth medium and transferred to a 25 cm^2^ plastic tissue culture flask. The cultures were incubated at 28° C without CO_2_. To assess the best culture medium, the seeding of embryonic tissues was carried out separately in the following culture media: L-15 (Gibco, Life Technologies, www.lifetechnologies.com), Grace/L-15 (Gibco), Eagle MEM (Gibco), MM (Mitsuhashi et al. 1964), VP12 (Varma and Pudney 1969), MM/VP12 (Varma and Pudney 1969), and Schneider (Sigma-Aldrich, www.sigmaaldrich.com). All media were supplemented with 20% foetal bovine serum (FBS) (Gibco) and a mixture of penicillin (100 units/mL), streptomycin (100 units/mL), and antimycotics (2.5 µg/mL Amphotericin B), and the pH of the media was adjusted to between 6.7 and 6.9. Additionally, the Eagle MEM medium was supplemented with 25 mM HEPES (N-2- hydroxy- ethylpiperazine-N′-2- ethanesulfonic acid) as a buffer system to be used without atmospheric CO_2_.

### Subculture

Primary cultured cells were subcultured with a 1:2 split ratio when the cells reached confluence in August of 2011; since that time, the cells have been maintained for over 21 passages. The first five subcultures were passaged at a ratio of 1:1 for each subculture, with an average duration of 20 days between each passage. Subcultures from the sixth to 11^th^ passages were carried out in a passage split ratio of 1:1 at 10-day intervals. Thereafter, the split ratio was increased gradually to 1:3 ratios every seven days. The cells were transferred to 25 cm^2^ flasks, which contained 5 mL of fresh culture medium. The incubation temperature was 28° C, and daily observations were performed using an inverted microscope.

### Morphological characteristics

Cell morphology and growth patterns were observed and photographed using an inverted microscope with phase contrast and a microphotographic system (Leica DMLI, http://www.leica-microsystems.com) in increments of 100 to 400X magnification. The most representative cells were photographed.

### Karyotype analysis

Metaphase chromosomes were prepared by taking primary cultures in a semi-confluent monolayer, and procedures from Schneider (1987) and Lee and Hou (1992) were applied with some modifications. Colchicine at a concentration of 0.6 µg/mL was added to the culture medium for a period of 3 hr. Then, the monolayer was removed, and the culture medium was centrifuged at 600 g for 10 min. The supernatant was discarded and 0.56% KCl was added to the precipitate. The mixture was stirred by flushing with a Pasteur pipette for 30 min. Then, the mixture was centrifuged again and Carnoy fixative (methanol and acetic acid, 3:1) was added for 15 min. Three successive washings with Carnoy were carried out. 1 mL of the cell suspension was dropped onto clean slides. The dried preparation was stained with 2% Giemsa. The best metaphases from the slides were selected and microphotographed.

### Analysis of isozyme patterns

Isozymatic phenotypes from four systems were examined: phosphoglucose isomerase (PGI-5.3.1.9), phosphoglucomutase (PGM- 2.7.5.1), malic dehydrogenase (ME), and phosphogluconate dehydrogenase (6-PGDH- 1.1.1.4.4). Isozymes were resolved by electrophoresis technique on cellulose acetate following the procedures described by Brown and Knudson (1980). The four isozymatic patterns from the cell cultures were compared with a cell line from *Lutzomyia longipalpis* (Lulo) (Rey et al. 2000) and with cell cultures from *Lucilia sericata* (Echeverry et al. 2009). The relative electrophoretic mobility (REM) was calculated using the formula: REM = e/a × 100, where “e” corresponds to the distance run in mm for each enzyme in the *S. magellanica* sample, and “a” corresponds to the distance run in mm for each enzyme in the Lulo sample. Migration was measured from the edge of the well where the sample had been applied to the corresponding band's midpoint (Zapata et al. 2005).

### Molecular characterization (RAPD-PCR)

Extraction, purification, and quantification of DNA from confluent monolayers of the *S. magellanica* and cell lines from Lulo and *L. spinicrassa* (Zapata et al. 2005) were performed according to a modified version of the method of Landry et al. (1993). The extraction of total DNA from *S. magellanica* adults were carried out using the procedure proposed by Coen et al. (1982). The PCR was performed using Invitrogen PCR ingredients (Life Technologies). PCR amplification was performed in 25 µL of a reaction mixture containing: 2.5 µL of Buffer A (10 x), 1 µL of dNTPs (10 mM), 1.25 µL of MgCl_2_ (50 mM), primer 1 (10 µM), 0.2 µL of Taq DNA polymerase (1 U/µl), 5 µL of DNA (10 ng/µL), and 14.05 µL of nuclease- free water (final volume of 25 µL per sample). Four synthesized primers were selected. The sequence for each of the primers was as follows: A2 = (5′-TGCCGAGCTG- 3′), A10 = (5′-ACGGCGTATG-3′), A20 = (5′-GTTGCGATCC-3′), and E07 = (5′- AGATGCAGCC-3′). The reaction mixture was placed in a thermal cycler (Esco, www.escoglobal.com), and the PCR was run at 94° C for 4 min (denaturation step), followed by 45 cycles consisting of 94° C for 1 min, 36° C for 1 min (annealing step), and 72° C for 2 min (extension step), with an additional 5 min at 72° C for the last cycle.

The reaction products were electrophoresed in a 1.4% agarose gel at 35 mA for 120 min. The agarose plate was then stained in 0.5 µg/mL ethidium bromide in TAE buffer (40 mM Tris, 20 mM acetic acid, 1 mM EDTA, pH 8.5) and photographed under ultraviolet light. Individual bands were scored as present or absent in the amplification profile of each sample (Stevens and Wall 1997).

Band patterns were compared using the similarity coefficient (SAB) of Ney and Li (1979), which is represented by the following formula: SAB = 2NAB / (NA + NB), where NA and NB correspond to the total number of bands shown by individual A and individual B, respectively, and NAB is the number of shared bands.

### Cryopreservation

For freezing and cryopreservation, monolayers' 80% confluents were detached, and the cells were adjusted to 5 × 106/mL with fresh medium (50%) containing 40% foetal bovine serum (Gibco) and 10% DMSO. When these cells were frozen, the time for the population doubling was about 30 hr. The suspension was dispersed into sterile cryotubes and refrigerated at 5° C, frozen overnight at -70° C, and then placed in liquid nitrogen for permanent storage.

## Results

Cell growth from embryonated egg explants in different flasks was first observed after about 48 hr. These cells grew in L-15, Grace/L15, and Schneider medium. There was no cell growth in MM, VP12, MM/VP12, or Eagle MEM medium. At the beginning cell growth was slow, but after few days the cell groups formed colonies attached to the surface of the flask. There was also proliferation of cells in suspension. Later, many of these cells were not viable, and others, which grew and proliferated to contribute to the formation of new cell colonies, adhered to the flask surface.

**Figure 1.**
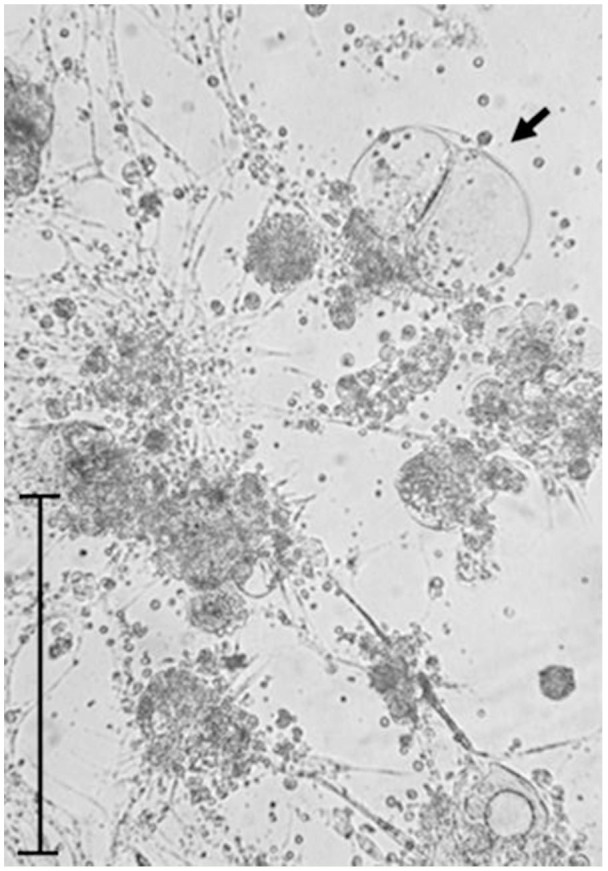
Growth pattern through vesicles observed in the primary cell cultures developed in the Schneider medium. Bar = 200 µm. High quality figures are available online.

**Figure 2.**
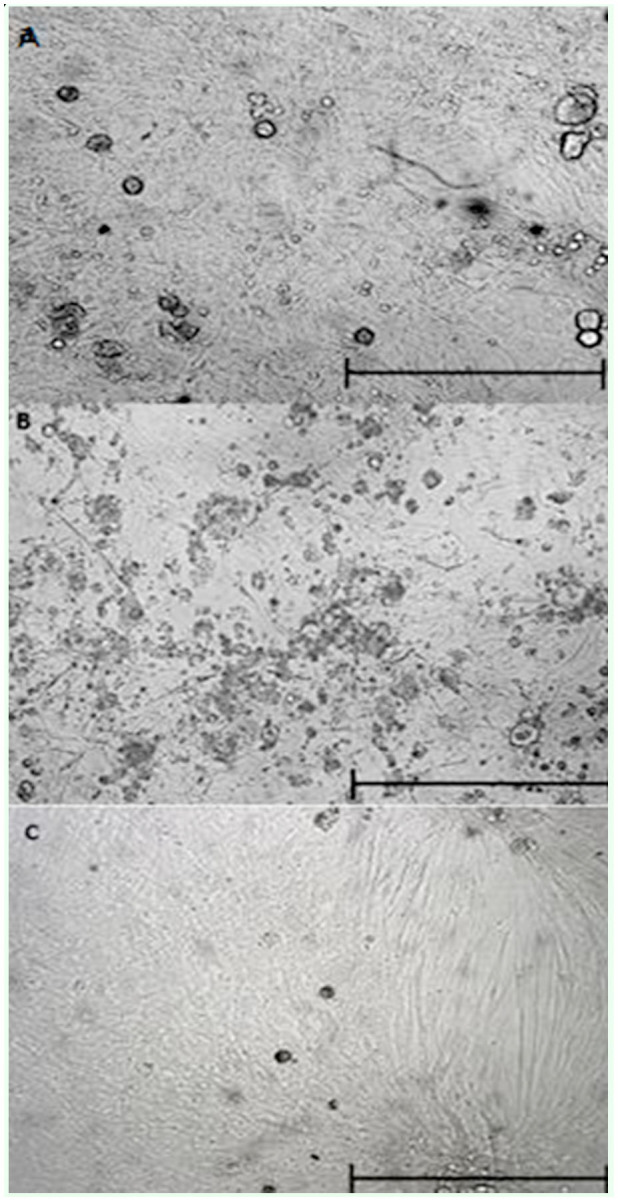
Confluent monolayers formed in three different culture media. (a) Grace/L15 medium eight days after the embryonic tissues were seeded (20×). (b) Schneider medium10 days after seeding tissues were explantted. (c) L15 medium 19 days after embryonic tissues were explanted. Bar = 200 µm. High quality figures are available online.

Many explanted tissue fragments were an important source of both cell migration and proliferation. Some tissue fragments had pulsating movements that were observed for more than two weeks. Moreover, many vesicles emerged from the extremities of the tissue fragments, and others were observed in suspension (Figure 1). An inverted microscope revealed that vesicles were formed by a monolayer of epithelial cells surrounding an empty space. Vesicle dissociation and rupture (which was initially spontaneous and later induced) became an important source of cells which then adhered to the culture flask surface and started to grow. During the following two weeks, there was formation of new cell colonies, which, together with others previously constituted, had a favourable evolution in growth until the formation of a confluent monolayer attached to the bottom of the flask. The time spent in the formation of the confluent monolayer in the three culture media where cell growth occurred was different among them. The Grace/L15 medium had the shortest period (8 days), followed by Schneider and L15, which had 10 and 19 days of duration respectively (Figure 2).

In general, the initiation of primary cell cultures in the three media where growth was present showed an evolution and proliferation that was extremely slow. The first successful subculture was carried out 8 days after the embryonic tissues were seeded. The number of passages obtained in each of culture media was the following: two in L-15, four in Grace/L-15, and in the Schneider medium 21 subcultures have been carried out up to date. In this medium, after the fifth passage, cell division increased in the cultures. At this time, the cells were subcultured at a ratio of 1:3 once per week. The viability of frozen cells was shown four months after freezing.

**Figure 3.**
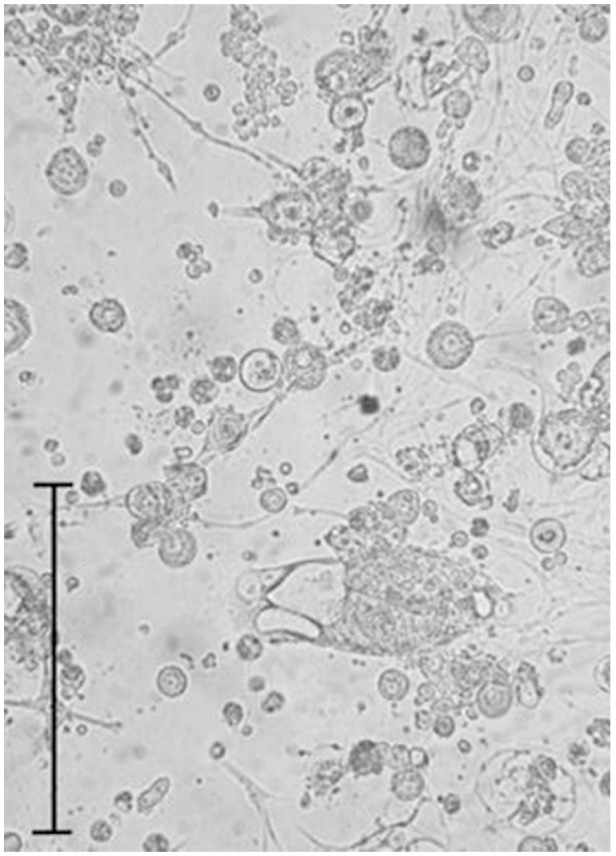
Predominant cell type in a cell culture developed in the Schneider medium from *Sarconsiopsis magellanica*, passage 18, with fibroblastoid cells. Bar = 200 µm. High quality figures are available online.

The *S. magellanica* cell cultures were initially composed of a heterogeneous cell population, being consistently elongated, spherical, small, presenting vesicles constituted by epithelioid cells, irregular, and occasionally giant shapes (Figure 3). However, in the confluent monolayer and in the subcultures, there was greater cell morphology uniformity, fibroblastoid types being predominant. In higher passages, the fibroblastoid cells sometimes had cytoplasmic fine filaments, which formed networks similar in appearance to nerve cells. This shape was also observed, in less proportion, in the initiation of the cell cultures.

**Figure 4.**
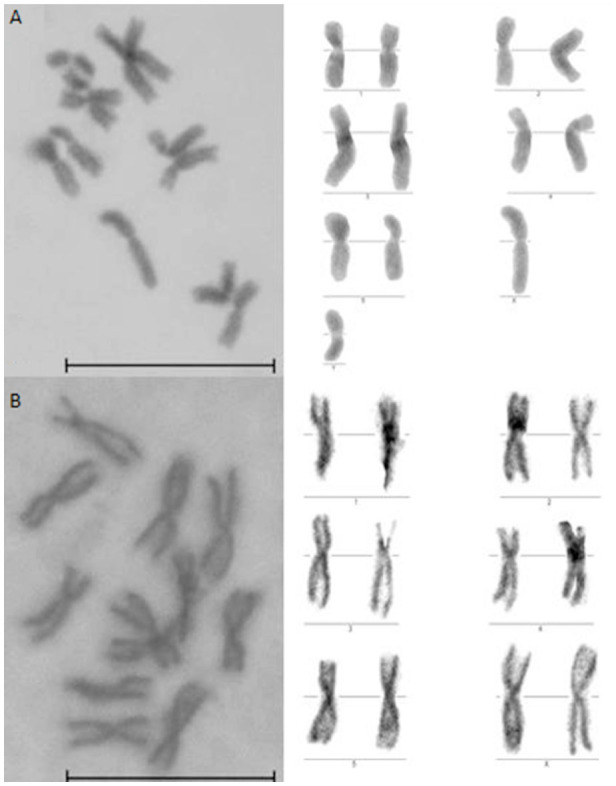
Giemsa-staining metaphase chromosome preparations from *Sarconesiopsis magellanica* cell cultures showing (a) the karyotype of the male and (b) the karyotype of the female. Bar = 25 µm. High quality figures are available online.

The metaphase arrays obtained from primary cultures and subcultures of *S. magellanica* exhibited a chromosomal number of 12 (Figure 4). Although the karyotype of this fly has never been reported, the set of 12 chromosomes was considered diploid because diploid cells of various species in the same family are reported to have 12 chromosomes. The karyotypic complement consisted of five pairs of somatic chromosomes and one sexual pair. This last pair was homomorphic (XX) in the female and heteromorphic (XY) in the male (Figure 4a, b). The Y chromosome was much shorter than the X chromosome.

**Figure 5.**
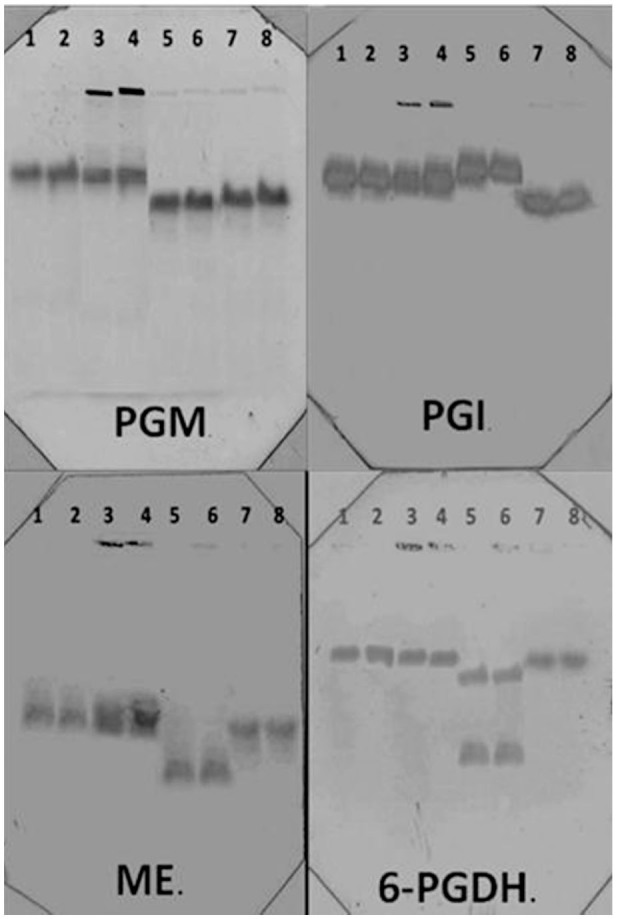
Isozyme patterns from four systems, PGM, PGI, ME, and 6-PGDH, which correspond to the following samples: lane 1 and 2, *Sarconesiopsis magellanica* cell cultures; lane 3 and 4, *Sarconesiopsis magellanica* larvae; lane 5 and 6,*Lucilia sericata* cell cultures; lane 7 and 8, *Lutzomyia longipalpis* cell line. High quality figures are available online.

The isozymatic phenotypes showed one band each for the four systems analyzed (PGI, PGM, ME-6, PGDH). These results coincided with those obtained from *S. magellanica* larvae samples simultaneously analyzed with the cell cultures. Although the *L. sericata* and Lulo cell lines had the same number of bands, excepting the 6-PGDH system, which had two bands for the *L. sericata* cell line, the electrophoretic mobilities were different. The isozymatic patterns for each of the different systems of the cell line can be observed in Figure 5.

**Figure 6.**
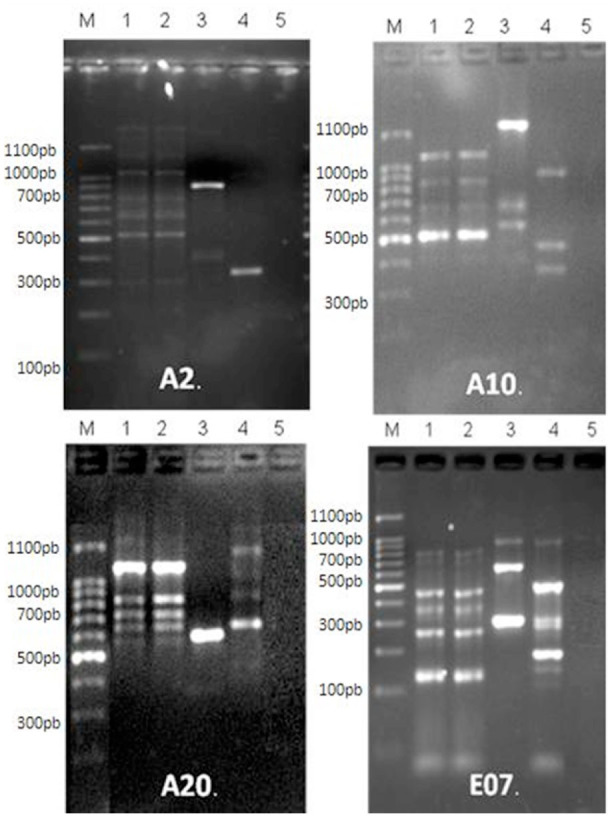
DNA (RAPD) profiles using four different primers: A2, A10, A20, and E07. Line M is the molecular weight marker. Line 1 profiles from the *Sarconesiopsis magellanica* cell line. Line 2 profiles from *S. magellanica* adults. Line 3 profiles from the *Lutzomyia spinicrassa* cell line. Line 4 profiles from the *Lutzomyia longipalpis* (Lulo) cell line. High quality figures are available online.

With the A2 primer, two DNA fragments were recorded for the *L. spinicrassa* and Lulo cell lines. Also, with this same primer, eight DNA fragments were obtained for cell cultures and adults from *S. magellanica* (Figure 6a). With the A10 primer, a profile of four DNA fragments was obtained from cell cultures and adults of *S. magellanica*, whereas three fragments were obtained from the *L. spinicrassa* and Lulo cell lines (Figure 6b). Using the A20 primer, a DNA fragment was obtained from Lulo and four were established from the *L. spinicrassa* cell line. Moreover, for this same primer, six fragments were obtained from cell cultures and adults derived from *S. magellanica* (Figure 6c). Finally, with the EO7 primer, the results showed six DNA fragments for the *S. magellanica* species (cell cultures and adults), three for Lulo, and five for the *L. spinicrassa* cell line (Figure 6d). Generally, the DNA fragments obtained covered a range from 100 to 1,100 bp.

The SAB values between DNA bands of the cell cultures and adults of *S. magellanica* were identical, but they were different compared with the cell lines previously established and maintained in our laboratory, which were mentioned above.

## Discussion

The main growth pattern observed in primary cultures of *S. magellanica* was similar to that reported by Zhang et al. (2012), as there was release and proliferation of cells from the tissue masses of both. Likewise, the vesicles observed in our cell cultures have also been registered in previous works (Rey et al. 2000; Segura et al. 2012), although both distribution and characteristics of the vesicles in the other research could be different**. One of the critical factors for successful initiation of primary cell cultures of insects was the incubation time of the eggs used in the embryonic tissue explants (Petcharawan et al. 2006)**, which should correspond to approximately two-thirds of the total time of embryo formation before the egg hatches (Segura et al. 2012). In the present work, this time was reached between 22 and 24 hr. However, it was also necessary to apply other additional temperatures to the embryonated eggs, first at 4° C and afterward at 37° C, prior to their use in tissue explants, which stimulated the cell division and produced the best results at the initiation of the primary cell cultures (Segura et al. 2012).

The presence of a pulsating movement observed in primary cultures of *S. magellanica* could be the result of the activity of muscle tissue, which is dependent on contractile proteins (Duce and Usherwood 1986; Segura et al. 2012). Moreover, its disappearance after the first three subcultures could be a consequence of an adaptive selection of the cells to the conditions established in the culture medium (Takahashi et al. 1980)

The time spent in cell growth and proliferation from embryo tissues of *S. magellanica* until the formation of the confluent monolayer was relatively short, similar to that reported by Segura et al. (2012). However, the registered differences among the three culture media, according to the time spent in the formation of the cell monolayer, were dependent on the process of adaptation and cell proliferation in response to the nutritional, physical-chemical, and environmental conditions established in the culture media (Zhang et al. 2011). Furthermore, the continuity of the cell subcultures registered from this species was achieved only in the Schneider medium because it included in its components two necessary nutrients for physiological processes of insects, trehalose and yeastolate, which probably promoted the adhesion, growth, and spreading of the cells on the surface of culture flasks (Zhang et al. 2011).

The varied cell morphology in the initiation of *S. magellanica* cultures frequently occurs in embryonic tissue from insects. These were adapted and grew in the selected media, giving origin to different cell shapes (Segura et al. 2012). However, these same cells in the monolayers and subcultures were changing into one just shape (Zhang et al. 2012), which is most likely the consequence of continuous cell adaptations. The cytoplasmic fine filaments observed in the first subculture were very similar to the description recorded by Echeverry et al. (2009) in the primary cell cultures derived from *L. sericata*, which is also a necrophagous fly belonging to the Calliphoridae family.

The *S. magellanica* cell culture chromosomal number coincided with the numbers reported for species of the Calliphoridae family. Although considerable morphological variation has been found in the karyotypes of the species in this family, the chromosomal number is very stable, with five autosomes and a heteromorphic sexual pair (Ullerich et al. 2006). The absence of polyploidy in the cell cultures derived from this fly showed that there has not been a culture transformation, which is different from the records in other studies where these chromosomal abnormalities were detected and consequently conduced the production of continuous growth cell lines (Petcharawan et al. 2006; Pan et al. 2010).

The isozymatic phenotypes of *S. magellanica* cultures compared with *L. sericata* and Lulo cell lines, two lines kept in our laboratory, were useful tolos for eliminating cross contamination of *S. magellanica* cultures with these cell lines. RAPD-PCR was used to characterize the cell cultures from *S. magellanica*, which permitted the authentication of the cells in accordance with the comparison done from adult samples of the same species. This technique could also distinguish the cell line derived from *S. magellanica* from others cell cultures of different species (e.g., *L. longipalpis and L. spinicrassa*)**. These results suggest that there was not a significant loss of genetic material and reflect the allelic diversity of the natural population of *S. magellanica* (Léry et al. 2003).

In conclusion, a cell line was obtained from embryonic tissues of *S. magellanica*. The morphological, karyological, isozymatic, and molecular characteristics of these cell cultures were determined. This cell line is a new *in vitro* model that will be used for preliminary studies of susceptibility to infections with arboviruses and as substrates for the development of the life cycle of some parasites.
